# Getting to Know the Gut Microbial Diversity of Metropolitan Buenos Aires Inhabitants

**DOI:** 10.3389/fmicb.2019.00965

**Published:** 2019-05-21

**Authors:** Fiorella Sabrina Belforte, Natalie Fernandez, Francisco Tonín Monzón, Ayelén Daiana Rosso, Sofía Quesada, María Cecilia Cimolai, Andrea Millán, Gloria Edith Cerrone, Gustavo Daniel Frechtel, Rémy Burcelin, Federico Coluccio Leskow, Alberto Penas-Steinhardt

**Affiliations:** ^1^Laboratorio de Genómica Computacional, Departamento de Ciencias Básicas, Universidad Nacional de Luján, Luján, Argentina; ^2^Programa de Estudios de Comunicación y Señalización Inter-Reino (PECSI), Departamento de Ciencias Básicas, Universidad Nacional de Luján, Luján, Argentina; ^3^Consejo Nacional de Investigaciones Científicas y Técnicas (CONICET), CABA, Buenos Aires, Argentina; ^4^Icahn School of Medicine at Mount Sinai, New York, NY, United States; ^5^Centro de Investigación, Docencia y Extensión en Tecnologías de la Información y las Comunicaciones (CIDETIC), Universidad Nacional de Luján, Luján, Argentina; ^6^Laboratorio de Diabetes y Metabolismo, Instituto de Inmunología, Genética y Metabolismo, Universidad de Buenos Aires-CONICET, CABA, Buenos Aires, Argentina; ^7^Institut National de la Santé et de la Recherche Médicale (INSERM), Toulouse, France; ^8^Université Paul Sabatier (UPS), Unité Mixte de Recherche (UMR) 1048, Institut des Maladies Métaboliques et Cardiovasculaires (I2MC), Team 2: ‘Intestinal Risk Factors, Diabetes, Dyslipidemia’, Toulouse, France; ^9^Fundación H.A. Barceló, Instituto Universitario de Ciencias de la Salud, CABA, Buenos Aires, Argentina

**Keywords:** microbiota, 16S rRNA, Buenos Aires, gut micobiome, westernized population

## Abstract

In recent years, the field of immunology has been revolutionized by the growing understanding of the fundamental role of microbiota in the immune system function. The immune system has evolved to maintain a symbiotic relationship with these microbes. The aim of our study was to know in depth the uncharacterized metagenome of the Buenos Aires (BA) city population and its metropolitan area, being the second most populated agglomeration in the southern hemisphere. For this purpose, we evaluated 30 individuals (age: 35.23 ± 8.26 years and BMI: 23.91 ± 3.4 kg/m^2^), from the general population of BA. The hypervariable regions V3-V4 of the bacterial 16S gene was sequenced by MiSeq-Illumina system, obtaining 47526 ± 4718 sequences/sample. The dominant phyla were Bacteroidetes, Firmicutes, Proteobacteria, Verrucomicrobia, and Actinobacteria. Additionally, we compared the microbiota of BA with other westernized populations (Santiago de Chile, Rosario-Argentina, United States-Human-microbiome-project, Bologna-Italy) and the Hadza population of hunter-gatherers. The unweighted UniFrac clustered together all westernized populations, leaving the hunter-gatherer population from Hadza out. In particular, Santiago de Chile’s population turns out to be the closest to BA’s, principally due to the presence of Verrucomicrobiales of the genus *Akkermansia*. These microorganisms have been proposed as a hallmark of a healthy gut. Finally, westernized populations showed more abundant metabolism related KEEG pathways than hunter-gatherers, including carbohydrate metabolism (amino sugar and nucleotide sugar metabolism), amino acid metabolism (alanine, aspartate and glutamate metabolism), lipid metabolism, biosynthesis of secondary metabolites, and sulfur metabolism. These findings contribute to promote research and comparison of the microbiome in different human populations, in order to develop more efficient therapeutic strategies for the restoration of a healthy dialogue between host and environment.

## Introduction

Higher multicellular organisms, such as humans, can be considered meta-organisms constituted by the individual’s own cells and their symbiotic commensal microbiota. With an estimated composition of 100 billion cells, human symbiotic microorganisms express 10 times more unique genes than those in the human genome (Ley et al., 2006). These complex communities of microorganisms, including bacteria, fungi, virus, and eukaryotic species not only enhance by several orders of magnitude the host’s enzymatic capacity, but also play a key role in controlling many aspects of the individual’s physiology.

In recent years, the field of immunology has been revolutionized by the growing understanding of the fundamental role of microbiota in the induction, training, and function of the host immune system (Belkaid and Hand, 2014). The immune system has evolved, to a large extent, as a means to maintain the symbiotic relationship between the host and these highly diverse and changing microbes. Under favorable conditions, the alliance between the immune system and the commensal microbiota allows the induction of protective responses against pathogens and the preservation of the regulatory pathways involved in the maintenance of tolerance to innocuous antigens (Chistiakov et al., 2014). However, profound changes in the environmental context could lead to the selection of a microbiota that lacks the resilience and diversity needed to establish balanced immune responses (Sommer et al., 2017). This suggests that environmental changes resulting from the modern lifestyle, such as antibiotic use, cesarean-section deliveries, excessive hygiene, and stress would account for the dramatic increase in autoimmune and chronic inflammatory disorders in geographical areas where the symbiotic relationship with the microbiota has been affected (Dominguez-Bello et al., 2010; Hall et al., 2017). Subclinical low-grade systemic inflammation has been associated with obesity, metabolic syndrome (MS) and type 2 diabetes mellitus (T2DM) (Schmidt et al., 2006). Several studies in animal models as well as in humans have shown that changes in the innate immune response are involved in the pathogenesis of these metabolic disorders (Hotamisligil, 2006; Cani et al., 2007). We have previously shown association between activity and expression levels of the toll-like receptor 4 (TLR4), a leucine-rich repeat molecule that plays a key role in the activation of innate immune response by recognizing conserved pathogen-associated molecular patterns such as lipopolysaccharides (LPS) and in metabolic disorders (Belforte et al., 2012; Penas-Steinhardt et al., 2012). The activation of TLR4 signaling induces up-regulation of inflammatory pathways related to the induction of insulin resistance. Others have highlighted the role of gut microbiota in the perpetuation of both insulin resistance and low-grade chronic inflammation (Petruzzelli and Moschetta, 2010), suggesting an alternative explanation for the increasing prevalence of insulin resistance related diseases.

There are several approaches under study to modulate the gut microbiota using external sources such as prebiotics, probiotics, antibiotics and fecal transplants (Balakrishnan and Taneja, 2018). Probiotics are live microbial species that modulate the gut microbiota in various ways: influencing immune homeostasis, regulating mucus secretion by gut epithelial cells, facilitating the production of a variety of nutrients such as vitamins, degrading toxic substances and producing antimicrobial compounds such as bacteriocins (Balakrishnan and Taneja, 2018). Consequently, regular probiotics consumption rapidly and reproducibly alters the human gut microbiome (David et al., 2014).

We are witnesses of an explosion of discoveries unveiling the role of gut microbial communities in health and disease states in humans (Garidou et al., 2015; Dickson, 2017). However, the normal composition of the microbiota of the human intestine is still under debate (van den Elsen et al., 2017). This provides a unique opportunity to develop a comprehensive exploration of human health from a multidisciplinary research strategy. Since Buenos Aires is the second most populated agglomeration in South America and the southern hemisphere, with a large genetic and cultural component of European immigration interacting with indigenous people, we propose to describe the fingerprint of its fecal microbiota. In this sense, it is fundamental to promote research defining the microbiome in different human populations to define future therapeutic strategies for the restoration of a healthy dialogue between host and environment, and thus to restore the health of the meta- Human organism.

## Materials and Methods

### Selection of Participants

We evaluated 30 individuals (age: 35.23 ± 8.26 years and BMI: 23.91 ± 3.4 kg/m^2^), from the general population of the Autonomous City of Buenos Aires (BA) and its metropolitan area, Argentina. The recruitment was performed in the Endocrinology Service of *Hospital de Clínicas “José de San Martín”* at Universidad de Buenos Aires, where clinic and anthropometric measurements were performed, blood samples were collected, and stool samples were received. Fecal samples were collected in household collection tubes delivered by the researchers responsible for the project. All participants signed written informed consent approved by the Ethics Committee of *Hospital Posadas*, Buenos Aires (according to the bioethical principles of the Declaration of Helsinki), approved by the Teaching, Research and Bioethics Committee of the Hospital Posadas (Ref CEPIC 119 EUPeS1/16).

#### Inclusion Criteria

Volunteer individuals from the general population of the city of Buenos Aires.

#### Exclusion Criteria

Individuals that have received antibiotic therapy in the last month, extreme diets (macrobiotic, vegan), gastrointestinal surgery (gastrectomy, bariatric surgery, colostomy), pregnancy, neoplasia, patients in therapy of renal replacement, transplanted or HIV patients.

### Phenotypic and Environmental Data

Clinical and biochemical data were evaluated as well as anthropometric measurements of the participants. Demographic characteristics, presence of environmental factors (type of delivery, feeding, consumption of probiotics, alcohol, smoking, physical activity), medication used for treatment according to the case, and family history of chronic inflammatory pathologies, and the preexistence of emerging diseases such as Obesity, T2DM, Arterial Hypertension, Dyslipidemia among others, were established by anamnesis.

### Sample Collection and DNA Extraction

During the interview participants were instructed in the collection method for stool sampling by receiving a standardized written protocol for the collection of approximately 5 g of stool in a sterile wide-mouth tube in DMSO/EDTA/saturated sodium chloride buffer (DESS buffer) (Gray et al., 2013). DESS Buffer has been shown to be highly effective in long-term DNA preservation at room temperature (Beknazarova et al., 2017). Tubes had a small spoon attached to the lid that facilitates the collection of the samples, which were suggested to be collected within 24 h prior to the next interview. Stool samples received were kept at -80°C until use. For each sample, DNA extraction was performed from 200 mg of feces using two different commercial kits following the instructions of the manufacturers: QIAamp DNA Stool Mini Kit (QIAGEN^®^) and Quick-DNA Soil (Zymo Research^®^). Nucleic acid concentration and purity were determined by 1% agarose gel electrophoresis and spectrophotometry on a NanoDrop ND-1000 (NanoDrop Technologies, Wilmington, DE, United States).

### Sequencing of Bacterial 16S rRNA Fragments

To amplify 16S rRNA gene fragments, 30 ng of purified DNA was used and the hypervariable regions V3 and V4 of the bacterial 16S gene were amplified using Bakt_341F/Bakt_805R primers contained Illumina overhang adapter sequences. Sequencing was performed using a MiSeq sequencer (Illumina^®^). Libraries were sequenced in paired-end mode, guaranteeing sequences of ∼550 bp long and ∼100.000 sequences per sample.

### Sequence Analysis and Comparison of Microbial Communities

De-multiplexed reads were quality trimmed using Trimmomatic (V0.36) (Bolger et al., 2014). Sequences generated were analyzed using quantitative insights into microbial ecology (QIIME) version 1.9.1 software package (Caporaso et al., 2010) for the identification of operative taxonomic units (OTUs) as well as taxonomic allocation and statistical analysis. To this end, the sequences obtained were compared with those deposited in Greengenes 13_8 database (DeSantis et al., 2006). Chimeric sequences were filtered using VSEARCH (Rognes et al., 2016). Phylum, Class, Family, and Gender were assigned to each reading with an *open_reference* OTU picking process (with sortmerna_coverage of 80%) (Kopylova et al., 2012). Additionally, to compare this dataset with other studies that targeted different 16S regions, closed-reference strategy was used. In order to normalize the subsequent bioinformatic analysis raw sequences from different dataset were used. In particular, we compare our population with the following 16S rRNA gene sequence data set: Healthy Cohort study-Production Phase 2(PP2) of human microbiome project (HMP) SRP002860 (The Human Microbiome Project Consortium, 2012); Rosario-Argentina (Carbonetto et al., 2016); Hadza population from Tanzania (Schnorr et al., 2014); Bologna-Italia (Schnorr et al., 2014) and Santiago-Chile (Schnorr et al., 2014; Fujio-Vejar et al., 2017). The sequences obtained in this study were forwarded and made available in Qiita database (ID 11839) and SRA: PRJNA503303. To account for multiple comparisons, *p*-values were adjusted by Benjamini–Hochberg FDR correction, applied separately at each taxonomic rank for a given comparison (Benjamini and Hochberg, 1995). To study microbial communities in different sample groups we used Unifrac algorithm (Lozupone and Knight, 2015), specially designed to describe and compare microbial communities based on massive sequencing of phylogenetic marker genes. In order to compare this dataset with other studies that targeted different 16S regions closed_reference strategy was used. Differences on beta diversity were assessed using ADONIS. Ellipses drawings for weighted UniFrac and unweighted UniFrac were performed using cov.dellipse from metRology R package.

Putative functional genes were predicted using PICRUSt (Phylogenetic Investigation of Communities by Reconstruction of Unobserved States) (Langille et al., 2013), which applies 16S rRNA gene to predict the abundance of functional genes by matching sample OTUs with reference genomes. In order to compare the relative abundance of the different taxa between groups, we performed linear discriminant analysis (LDA) effect implemented in LEfSe (Segata et al., 2011).

## Results

### Buenos Aires General Population Data

Buenos Aires and its metropolitan area constitute a megalopolis, being the second most populated agglomeration in South America and the southern hemisphere with 12.806.866 of inhabitants. According to the last census (2010), the average age of the Autonomous City of Buenos Aires (BA) is 39.66 years. As reported by the “Third National Survey of Risk Factors for Non-transmissible Diseases” (NSRF) of the National Ministry of Health performed in 2013 (Tercera encuesta nacional de factores de riesgo para enfermedades no transmisibles, 2013), the average daily servings of fruits or vegetables consumed per person in the general population of BA was 2.1, being below the amount recommended by WHO (five daily servings of fruits and/or vegetables). The highest averages of daily fruit or vegetable consumption were recorded in the oldest age group (2.4), in the highest education level (2.1) and higher income level. In addition, the prevalence of overweight individuals was 32% (IC 95% 27.6 – 36.8), the prevalence of high cholesterol among those who were once controlled (population 18 years and older) was 27.9% (IC 95% 24.0 – 32.1), and 8.2% marked the prevalence of diabetes in the total adult population (IC 95% 6.4 – 10.5).

We evaluated 30 individuals (Female/male: 15/15; age: 35.23 ± 8.26 years and BMI: 23.91 ± 3.4 kg/m^2^), from the general population of BA (Table 1).

**Table 1:** Anthropometric and biochemical data of the 30 volunteers.

	Mean	SD
Female/male	15/1	
Age (years)	35.23	8.26
Weight (kg)	69.48	12.82
Height (m)	170.17	9.73
Body mass index (kg/m^2^)	23.91	3.40
Glycemia (mg/dl)	80.48	11.33
Triglycerides (mg/dl)	75.24	20.48
Total cholesterol (mg/dl)	168.24	29.80
High density lipoproteins (mg/dl)	55.10	17.06
Low density lipoproteins (mg/dl)	168.23	29.80


### Samples Collection and Storage

Considering that several methods could be used for stool collection and storage, we standardized sample collection and storage protocol prior processing. In our population based study, participants were instructed in a home collection method for stool sampling, proved to be as effective as freshly collected native stool (Schultze et al., 2014). This strategy comes from the preference of most participants who do not agree to collect the stool samples elsewhere. Therefore, each participant received a written protocol for easy stool collection, consisting in introducing approximately 5 g of stool in a sterile wide-mouth tube with DESS buffer (Gray et al., 2013). This solution preserves the samples both at room temperature and in sub-zero conditions, avoiding freezing and cell damage. Additionally, DESS buffer is a non-proprietary solution that has been used for DNA preservation (Gaither et al., 2010) as it guarantees long term storage at room temperature (up to 24 weeks), and has been used to preserve bacterial DNA from coral mucus swabs for up to 4.5 months (May, 2011). Additionally, given that it is known that freezing and thawing the samples could generate OTUs identification bias (Fouhy et al., 2015), this buffer represents an efficient alternative strategy for adequate sampling and preservation until processing.

### DNA Extraction Method

DNA isolation protocol and the targeted 16S rRNA gene region sequence chosen for analysis have been shown to be critical for gut microbiota assessment (Voigt et al., 2015), limiting the ability to compare different human microbiome datasets. In other to evaluate possible biases in the 16S profiles related to DNA extraction method, we compare QIAamp DNA Stool Mini (QG) and Quick-DNA Soil (ZR) commercial kits. DNA extraction was performed from 200 mg of feces using both QG and ZR for each sample. Fecal DNA extraction using QC and ZR yielded an average of 49.59 ± 21.41 and 24.85 ± 20.75 μg/mL DNA, respectively. The hypervariable regions V3-V4 of bacterial 16S gene was sequenced using MiSeq-Illumina system, and an average of 47526 ± 4718 sequences per sample were obtained. Rarefaction plots reach an asymptotic state, indicating that the sequence depth was sufficient to represent the bacterial community richness and diversity by both methods (data not shown). In this sense, when we compare alpha diversity (Chao1 index) based on a two-sample *t*-test, there are not significant differences between QG and ZR kits (*p* = 0.069). We also found no significant differences when comparing beta diversity between different purification kits, *p* = 0.97 for weighted UniFrac and unweighted UniFrac *p* = 0.294 (ADONIS).

Comparing the principal phyla detected using both DNA extraction Kits, we found that the choice of DNA extraction method has an impact on the observed community structure. Using the QG kit, the dominant phyla were Bacteroidetes (47.7 ± 8.9%), Firmicutes (37.2 ± 8.45%), Proteobacteria (8.5 ± 6.7%), Verrucomicrobia (2.5 ± 2.8%) and Actinobacteria (1.3 ± 2.2%), while the principal phyla found using ZR kit were Firmicutes (45.5 ± 8.8%), and Bacteroidetes (40.0 ± 7.8%), followed by Proteobacteria (6.1 ± 5.4%), Verrucomicrobia (2.5 ± 3.0%), and Actinobacteria (3.1 ± 2.2%). In this sense, QG protocol, without the bead-beating step, resulted in a significantly low abundance in Gram-positive phyla, Firmicutes (*p* = 0.015); Actinobacteria (*p* = 9.29 × 10^-5^) and overestimated Bacteroidetes phyla (*p* = 0.023) FDR adjusted *p*-values. These results, in accordance with previous studies (Guo and Zhang, 2013; Wesolowska-Andersen et al., 2014), show that DNA extraction method has an impact on the observed community structure. In particular, the first step of DNA extraction bead-beating disruption and/or lysis of the bacterial membranes can be expected to be biased for specific bacterial taxa due to differences in cell wall structure and integrity.

Since several studies have proposed the Firmicutes to Bacteroidetes ratio (F/B:ratio) as a gauge of short-chain fatty acids (SCFA) production and the overall gut microbiota balance any bias in these parameters must be taken into account (Rodríguez-Carrio et al., 2017; Garcia-Mantrana et al., 2018). In particular, QG protocol showed an increase in the abundance of Bacteroidetes phylum, main acetate and propionate producers, and a decrease in Firmicutes butyrate-producers (Macfarlane and Macfarlane, 2003). It is important to remark that these changes impact notably the F/B: ratio, being 0.77 for QG kit vs. 1.13 for ZR one. However, it is not possible to determine which DNA extraction method best represents the overall microbial community.

### Analysis of BA Microbiota

Our data is consistent with previous observations showing that the regular intake of yogurt and other fermented products promotes a healthy gut microbiota (Lisko et al., 2017; Marco et al., 2017; Suzuki et al., 2017). In this sense we found significant differences in alpha diversity Chao1 index (*p* = 0.012) when comparing microbial diversity between subjects that habitually consume probiotics with those who do not (considering both extraction kits).

**FIGURE 1:**
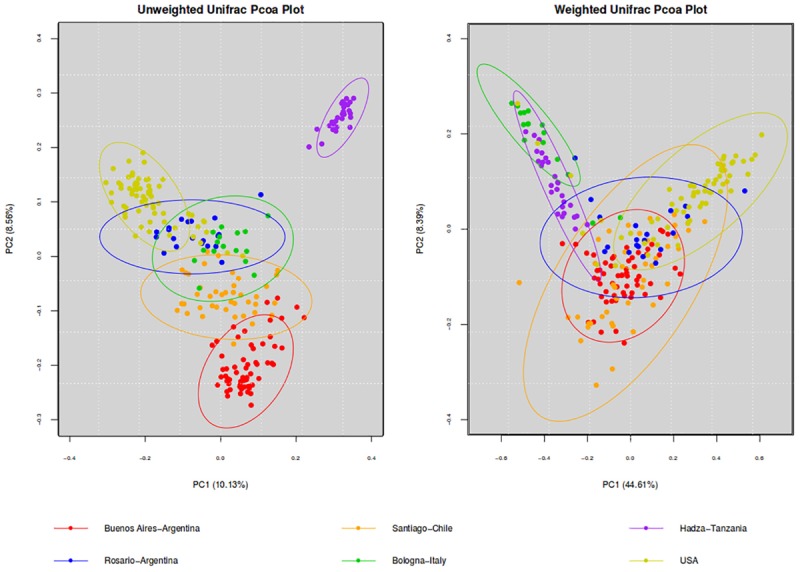
PCoA of beta diversity values (unweighted and weighted Unifrac distances). Comparison of the gut microbiota from individuals of BA and other geographic locations. Ellipses were showed confidence interval of 95%.

To further analyze our results, we compared the microbiota of BA with other westernized populations (Santiago de Chile, Rosario-Argentina, United States-Human-microbiome-project, Bologna-Italy) and the Hadza population of hunter-gatherers (Table 2). To understand differences in the composition of microbial communities’ diversity among environments was compared. Beta diversity (considering weighted and unweighted UniFrac distances) was analyzed in order to compare the differences between microbial compositions among different populations (Figure 1). Differences on beta diversity values between habitats, countries, cities, 16S region amplified, purification kit employed, and platform were evaluated (ADONIS). Although all the comparisons were statistically significant (*p* < 0.001), the *R*^2^-value indicates that approximately 43% (weighted) and 23% (unweighted) of the variation effect is produced by the city of origin, being the most important variable studied. The unweighted UniFrac (a qualitative measure that estimates the distance between two communities) clustered the westernized populations together, leaving the hunter-gatherer population from Hadza out. In particular, Santiago de Chile’s population turns out to be the closest to BA’s.

**Table 2:** Data-sets included in the analysis.

Platform	Country	City	Purification _kit	Region	Center_name	Habitat	Publication
454 LifeSciences-Roche	Tanzania	Tanzania-Lake Eyasi	QIAmp DNA Stool Mini Kit (Qiagen)	V4	Metabolism Anthropometry and Nutrition Laboratory Department of Anthropology University of Nevada.	Hunter-Gathe rer	Gut microbiome of the Hadza hunter-gatherer s_doi: 10.1038/n comms4654
454 LifeSciences-Roche	Italy	Italy-Bologna	QIAmp DNA Stool Mini Kit (Qiagen)	V4	Metabolism Anthropometry and Nutrition Laboratory Department of Anthropology University of Nevada.	Westernized	Gut microbiome of the Hadza hunter-gatherer s_doi: 10.1038/n comms4681
MiSeqSytem -Illumina	Argentina	Argentina-Buenos_Aires	QIAmp DNA Stool Mini Kit (Qiagen) and Quick-DNA Soil (Zymo Research).	V3-V4	GeC-UNLu	Westernized	Buenos Aires microbiome
MiSeqSytem -Illumina	Chile	Chile-Santiago	QIAmp DNA Stool Mini Kit (Qiagen)	V3-V4	Centro de Genética y Genómica	Westernized	The Gut Microbiota of Healthy Chilean Subjects Reveals a High Abundance of the Phylum Verrucomicrobi a: https://doi.org/10.3389/fmicb.2017.01221
454 LifeSciences-Roche	Argentina	Argentina-Rosario	QIAmp DNA Stool Mini Kit (Qiagen)	V1-V3	INDEAR	Westernized	Human Microbiota of the Argentine Population- A Pilot Study: https://doi.org/10.3389/fmicb.2016.00051
454 LifeSciences-Roche	United States	United States	MoBio Powersoil Kit	V3-V5	Human Microbiome Project (HMP)	Westernized	Healthy Cohort study:Productio n Phase 2(PP2)-SRP002 860


**FIGURE 2:**
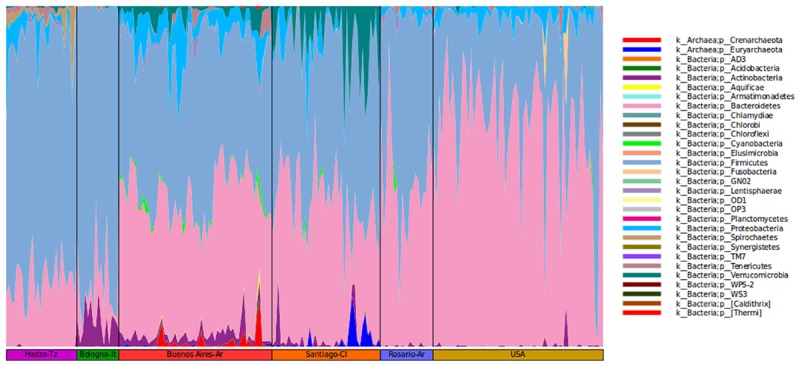
Community composition of gut microbiome from each geographic location shown at the phylum level.

It is relevant to highlight that the microbiota of BA subjects stands out for the presence of Verrucomicrobia of the genus *Akkermansia* (1.2%). The mucus-degrading bacterium *Akkermansia* muciniphila is the only identified member of this genus and was also found in the Chilean population (Fujio-Vejar et al., 2017). This microorganism has been proposed by others as a hallmark of a healthy gut due to its anti-inflammatory and immunostimulant properties and its ability to improve gut barrier function, insulin sensitivity and endotoxemia (Collado et al., 2007; Bland, 2016). However, some studies report the induction of pro-inflammatory responses triggered by *Akkermansia* contributing to an increase of inflammation during infection in Multiple Sclerosis patients (Cekanaviciute et al., 2017). As proposed by Fujio-Vejar et al., these results could serve as a baseline for the characterization of dysbiosis associated with various diseases (Fujio-Vejar et al., 2017; Figure 2).

**FIGURE 3:**
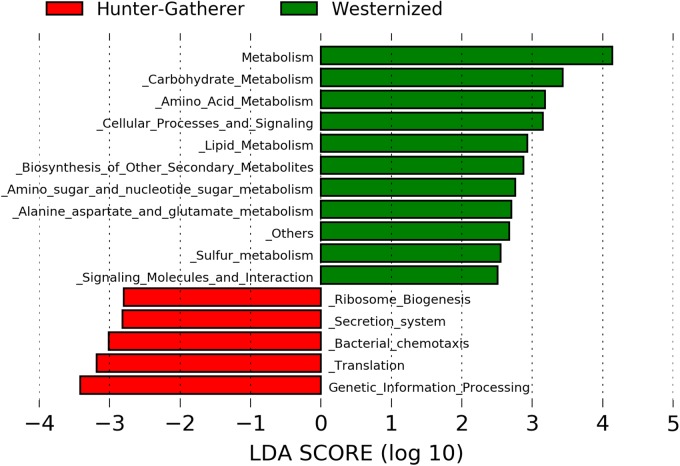
Linear discriminant analysis (LDA) effect size (LEfSe) analysis of the differentially abundant KEEG pathways in hunter-gatherers vs. westernized population. The threshold of the logarithmic LDA score was 2.0.

Putative functional genes were predicted using PICRUSt which applies 16S rRNA gene to predict the abundance of functional KEEG pathways. The LDA effective size (LEfSe) was performed to identify KEEG pathways with statistically differential abundance between hunter-gatherers and westernized population. The results show that metabolism related pathways were more abundant in westernized populations than in hunter-gatherers. In particular, carbohydrate metabolism (amino sugar and nucleotide sugar metabolism), amino acid metabolism (alanine, aspartate and glutamate metabolism), lipid metabolism, biosynthesis of secondary metabolites, and sulfur metabolism showed a LDA score greater than 2.0 (Figure 3).

## Discussion

The intestine harbors a sophisticated ecosystem of microbial communities, exerting vital metabolic functions that contribute to the recovery of nutrients and energy from non-digestible substrates. Microbial colonization is essential for the normal development of the immune system, regulating the homeostasis between environmental antigenic load and immune response. In susceptible individuals, imbalance could result into pathologies of immune dysregulation, including chronic inflammatory bowel diseases and metabolic syndrome, in which the immune system overreacts to non-harmful microbial antigens (Ananthakrishnan et al., 2017; Burcelin, 2017). Although there are new molecular technologies, the normal composition of the microbiota of the human intestine is still under debate (van den Elsen et al., 2017). Many environmental factors, such as geographic localization, household characteristics and circumstances as delivery method, antibiotic use, breastfeeding or diet in the early life, are conditioning factors for microbial colonization of the intestine. In permissive genetic backgrounds, environmental reprogramming of microbiota can trigger conditions for the establishment of an imbalanced immune response (Ussar et al., 2015).

The number of public human metagenomes shows a marked bias toward the north hemisphere. Analyzing the total metagenomic data available today, the northern hemisphere outnumbers that from the south by 200 times. Since Buenos Aires is the second most populated agglomeration in South America and the southern hemisphere, with a large genetic and cultural component of European immigration interacting with indigenous people, we propose to describe the fingerprint of its fecal microbiota. Given the hypothesis that there are differences between the intestinal microbiota composition of healthy and diseased subjects, we proposed to draw a baseline study of the uncharacterized gut microbiota from the general population of BA for future comparisons with any further studies.

During the interviews, participants were instructed in the home collection method of stool sampling, which was suggested to be collected within the 24 h prior to the next interview. Stool samples were remitted and stored at -80°C until use. In order to prevent changes in fecal microbiota, 16S gene sequencing profiles related to ambient temperature (Roesch et al., 2009; Carroll et al., 2012) or sub-zero storage conditions (Bahl et al., 2012; Fouhy et al., 2015) sampling was performed in a sterile wide-mouth tube with DESS buffer, since performing the nucleic acid extraction of samples immediately after collection was impractical. DESS buffer showed to be particularly efficient for sample transfer and storage at -80°C until processing, and it allowed the adequate conservation of the samples.

DNA extraction could also add to methodological biases in PCR amplification due to the presence of contaminants that could inhibit the reaction or the efficiency in DNA purification. Additionally, disruption and/or lysis of the bacterial membranes could be another source of bias, as it may favor the presence of specific bacterial taxa due to differences in cell wall structure and integrity (Sinha et al., 2016; Vogtmann et al., 2017). We have chosen two worldwide used commercial kits in order to evaluate microbiota profile sampling. Indeed, we observed differences in two of the most abundant phyla, Firmicutes and Bacteroidetes, which were differently affected, showing the effect of the extraction methods between Gram-positive and Gram-negative bacterial membranes. Interestingly, when we compare alpha diversity no significant differences between kits were observed. We also found no significant differences when comparing beta diversity, for weighted UniFrac and unweighted UniFrac, between purification methods. Although principal phyla were detected using both kits, we found that the choice of DNA extraction method has an impact on the observed community structure. In particular, these notably alter the F/B ratio, not allowing determining which DNA extraction method is the best to represent the microbial community. In addition, when we compare the microbiota of BA with other populations, we observed differences on beta diversity values between habitats, countries, cities, 16S region amplified, Purification Kit employed and Platform. Although all the comparisons were statistically significant, the *R*^2^-value indicates that approximately 43% (weighted) and 23% (unweighted) of the variation effect is produced by the city of origin, being the most important variable studied. However, when taxa abundance, such as F/B ratio, is analyzed between different studies the purification method must be taken into account. In this sense, future gold standard purification method should be developed to sort this bias to allow accurate conclusions.

In order to evaluate similarities and differences within other populations, we compare our microbiome with other westernized populations and the Hadza population of hunter-gatherers. The unweighted UniFrac, that estimate the distance between two communities, clustered together all westernized populations leaving the hunter-gatherer population from Hadza out. This Qualitative measure which uses only the presence/absence of data is most informative when communities differ primarily by which microorganism can live in them (Lozupone et al., 2007). Additionally, unweighted UniFrac can better detect effects of different founding populations, such as the source of bacteria that first colonize the gut of newborns and the effects of restrictive factors for microbial growth. In contrast, quantitative measures that account for the relative abundance of microbial lineages can reveal the effects of more transient factors.

In this sense, the microbiota of BA subjects stands out for the presence of the mucus-degrading bacterium *Akkermansia* muciniphila, the only identified member of its genus. This microorganism, also found in the Chilean population, has been proposed as a hallmark of a healthy gut due to its anti-inflammatory and immunostimulant properties and its ability to improve gut barrier function, insulin sensitivity and endotoxemia (Collado et al., 2007; Fujio-Vejar et al., 2017).

Finally, westernized populations showed more abundant metabolism related KEEG pathways than hunter-gatherers, including carbohydrate metabolism (amino sugar and nucleotide sugar metabolism), amino acid metabolism (alanine, aspartate and glutamate metabolism), lipid metabolism, biosynthesis of secondary metabolites and sulfur metabolism. This could be due to feeding habits since Hadza diet consists only of wild foods that fall into five main categories: meat, honey, baobab, berries and tubers (Schnorr et al., 2014).

Environmental factors, such as lifestyle, diet, the use of antibiotics, and the host genotype affects the human microbiome. It is important to point out that most of the available studies evaluate European, Asian, and North American populations that significantly differ both in the genetic background and in diverse environmental factors with BA population. On the contrary, very little is known about the microbiome of the Argentinean population, in particular there is currently only a pilot study of the population of Rosario city, Santa Fe (Carbonetto et al., 2016). It was then essential to know in depth the characteristic metagenome of the residents of BA and its metropolitan area which constitute a megalopolis, being the second most populated agglomeration in South America and the southern hemisphere with 12.806.866 of inhabitants.

In this sense, it is crucial to identify specific groups of the intestinal microbiota that could affect the susceptibility and/or severity of disease by stimulating chronic inflammatory responses (Surana and Kasper, 2017). Diversity among environments, genotypes and, therefore, microbial factors of the intestinal flora between human populations make it difficult to find ubiquitous biomarkers worldwide. Knowing the microbiota composition of the general BA population provides a framework to have a local reference that will allow establishing future correlations between health and disease. This is essential, not only for evaluating changes in the gut microbiota regarding different social habits and clinical features, but also to contribute to the development of personalized nutritional and pharmacological strategies to promote a healthy intestinal flora.

## Ethics Statement

This study was carried out in accordance with the recommendations of the Academic and Bioethics Committee of the National University of Luján with written information consent from all subjects. All subjects gave written informed consent in accordance with the Declaration of Helsinki. The protocol was approved by the Academic and Bioethics Committee of the National University of Luján (Ref TRI-LUJ: 0005667/2018).

## Author Contributions

FB and AP-S designed the study. NF, GC, and GF performed the recruitment of the volunteers. NF, AR, SQ, and AM collected the stool samples and the extraction of fecal bacterial DNA. FB, FTM, and AP-S processed the raw sequences and performed the bioinformatic and statistical analysis. FB, MC, RB, FCL, and AP-S analyzed the results and wrote the manuscript.

## Conflict of Interest Statement

The authors declare that the research was conducted in the absence of any commercial or financial relationships that could be construed as a potential conflict of interest.

## References

[B1] Ananthakrishnan A. N. Bernstein C. N. Iliopoulos D. Macpherson A. Neurath M. F. Ali R. A. R. (2017). Environmental triggers in IBD: a review of progress and evidence. *Nat. Rev. Gastroenterol. Hepatol.* 15 39–49. 10.1038/nrgastro.2017.136 29018271

[B2] Bahl M. I. Bergström A. Licht T. R. (2012). Freezing fecal samples prior to DNA extraction affects the Firmicutes to Bacteroidetes ratio determined by downstream quantitative PCR analysis. *FEMS Microbiol. Lett.* 329 193–197. 10.1111/j.1574-6968.2012.02523.x 22325006

[B3] Balakrishnan B. Taneja V. (2018). Microbial modulation of the gut microbiome for treating autoimmune diseases. *Expert Rev. Gastroenterol. Hepatol.* 12 985–996. 10.1080/17474124.2018.1517044 30146910

[B4] Beknazarova M. Millsteed S. Robertson G. Whiley H. Ross K. (2017). Validation of DESS as a DNA preservation method for the detection of strongyloides spp. in *Canine Feces*. *Int. J. Environ. Res. Public Health* 14:E624. 10.3390/ijerph14060624 28598404 PMC5486310

[B5] Belforte F. S. Coluccio Leskow F. Poskus E. Penas Steinhardt A. (2012). Toll-like receptor 4 D299G polymorphism in metabolic disorders: a meta-analysis. *Mol. Biol. Rep.* 40 3015–3020. 10.1007/s11033-012-2374-5 23275193

[B6] Belkaid Y. Hand T. W. (2014). Role of the microbiota in immunity and inflammation. *Cell.* 157 121–141. 10.1016/j.cell.2014.03.011 24679531 PMC4056765

[B7] Benjamini Y. Hochberg Y. (1995). Controlling the false discovery rate: a practical and powerful approach to multiple testing. *J. R. Stat. Soc. Ser. B Stat. Methodol.* 57 289–300. 10.1111/j.2517-6161.1995.tb02031.x

[B8] Bland J. (2016). Intestinal microbiome, and medical nutrition therapy. *Integr. Med.* 15 14–16. PMC5145007 27980489

[B9] Bolger A. M. Lohse M. Usadel B. (2014). Trimmomatic: a flexible trimmer for Illumina sequence data. *Bioinformatics* 30 2114–2120. 10.1093/bioinformatics/btu170 24695404 PMC4103590

[B10] Burcelin R. (2017). [Gut microbiota and immune crosstalk in metabolic disease]. *Biol. Aujourdhui* 211 1–18. 28682223 10.1051/jbio/2017008

[B11] Cani P. D. Amar J. Iglesias M. A. Poggi M. Knauf C. Bastelica D. (2007). Metabolic endotoxemia initiates obesity and insulin resistance. *Diabetes* 56 1761–1772. 17456850 10.2337/db06-1491

[B12] Caporaso J. G. Kuczynski J. Stombaugh J. Bittinger K. Bushman F. D. Costello E. K. (2010). QIIME allows analysis of high-throughput community sequencing data. *Nat. Methods* 7 335–336. 20383131 10.1038/nmeth.f.303 PMC3156573

[B13] Carbonetto B. Fabbro M. C. Sciara M. Seravalle A. Méjico G. Revale S. (2016). Human microbiota of the argentine population- a pilot study. *Front. Microbiol.* 7:51. 10.3389/fmicb.2016.00051 26870014 PMC4733923

[B14] Carroll I. M. Ringel-Kulka T. Siddle J. P. Klaenhammer T. R. Ringel Y. (2012). Characterization of the fecal microbiota using high-throughput sequencing reveals a stable microbial community during storage. *PLoS One* 7:e46953. 10.1371/journal.pone.0046953 23071673 PMC3465312

[B15] Cekanaviciute E. Yoo B. B. Runia T. F. Debelius J. W. Singh S. Nelson C. A. (2017). Gut bacteria from multiple sclerosis patients modulate human T cells and exacerbate symptoms in mouse models. *Proc. Natl. Acad. Sci. U.S.A.* 144 10713–10718. 10.1073/pnas.1711235114 28893978 PMC5635915

[B16] Chistiakov D. A. Bobryshev Y. V. Kozarov E. Sobenin I. A. Orekhov A. N. (2014). Intestinal mucosal tolerance and impact of gut microbiota to mucosal tolerance. *Front. Microbiol.* 5:781 10.3389/fmicb.2014.00781 PMC4292724 25628617

[B17] Collado M. C. Derrien M. Isolauri E. de Vos W. M. Salminen S. (2007). Intestinal integrity and Akkermansia muciniphila, a mucin-degrading member of the intestinal microbiota present in infants, adults, and the elderly. *Appl. Environ. Microbiol.* 73 7767–7770. 10.1128/aem.01477-07 17933936 PMC2168041

[B18] David L. A. Maurice C. F. Carmody R. N. Gootenberg D. B. Button J. E. Wolfe B. E. (2014). Diet rapidly and reproducibly alters the human gut microbiome. *Nature* 505 559–563. 10.1038/nature12820 24336217 PMC3957428

[B19] DeSantis T. Z. Hugenholtz P. Larsen N. Rojas M. Brodie E. L. Keller K. (2006). Greengenes, a chimera-checked 16S rRNA gene database and workbench compatible with ARB. *Appl. Environ. Microbiol.* 72 5069–5072. 10.1128/aem.03006-05 16820507 PMC1489311

[B20] Dickson I. (2017). Gut microbiota: diagnosing IBD with the gut microbiome. *Nat. Rev. Gastroenterol. Hepatol.* 14:195. 10.1038/nrgastro.2017.25 28250469

[B21] Dominguez-Bello M. G. Costello E. K. Contreras M. Magris M. Hidalgo G. Fierer N. (2010). Delivery mode shapes the acquisition and structure of the initial microbiota across multiple body habitats in newborns. *Proc. Natl. Acad. Sci. U.S.A.* 107 11971–11975. 10.1073/pnas.1002601107 20566857 PMC2900693

[B22] Fouhy F. Deane J. Rea M. C. O’Sullivan Ó Paul Ross R. O’Callaghan G. (2015). The effects of freezing on faecal microbiota as determined using miseq sequencing and culture-based investigations. *PLoS One* 10:e0119355. 10.1371/journal.pone.0119355 25748176 PMC4352061

[B23] Fujio-Vejar S. Vasquez Y. Morales P. Magne F. Vera-Wolf P. Ugalde J. A. (2017). The gut microbiota of healthy chilean subjects reveals a high abundance of the phylum verrucomicrobia. *Front. Microbiol.* 8:1221. 10.3389/fmicb.2017.01221 28713349 PMC5491548

[B24] Gaither M. R. Szabó Z. Crepeau M. W. Bird C. E. Toonen R. J. (2010). Preservation of corals in salt-saturated DMSO buffer is superior to ethanol for PCR experiments. *Coral Reefs* 30 329–333. 10.1007/s00338-010-0687-1

[B25] Garcia-Mantrana I. Selma-Royo M. Alcantara C. Collado M. C. (2018). Shifts on gut microbiota associated to mediterranean diet adherence and specific dietary intakes on general adult population. *Front. Microbiol.* 9:890. 10.3389/fmicb.2018.00890 29867803 PMC5949328

[B26] Garidou L. Pomié C. Klopp P. Waget A. Charpentier J. Aloulou M. (2015). The gut microbiota regulates intestinal CD4 T cells expressing RORγt and controls metabolic disease. *Cell Metab.* 22 100–112. 10.1016/j.cmet.2015.06.001 26154056

[B27] Gray M. A. Pratte Z. A. Kellogg C. A. (2013). Comparison of DNA preservation methods for environmental bacterial community samples. *FEMS Microbiol. Ecol.* 83 468–477. 10.1111/1574-6941.12008 22974342

[B28] Guo F. Zhang T. (2013). Biases during DNA extraction of activated sludge samples revealed by high throughput sequencing. *Appl. Microbiol. Biotechnol.* 97 4607–4616. 10.1007/s00253-012-4244-4 22760785 PMC3647099

[B29] Hall A. B. Tolonen A. C. Xavier R. J. (2017). Human genetic variation and the gut microbiome in disease. *Nat. Rev. Genet.* 18 690–699. 10.1038/nrg.2017.63 28824167

[B30] Hotamisligil G. S. (2006). Inflammation and metabolic disorders. *Nature* 444 860–867. 17167474 10.1038/nature05485

[B31] Kopylova E. Noé L. Touzet H. (2012). SortMeRNA: fast and accurate filtering of ribosomal RNAs in metatranscriptomic data. *Bioinformatics* 28 3211–3217. 10.1093/bioinformatics/bts611 23071270

[B32] Langille M. G. I. Zaneveld J. Caporaso J. G. McDonald D. Knights D. Reyes J. A. (2013). Predictive functional profiling of microbial communities using 16S rRNA marker gene sequences. *Nat. Biotechnol.* 31 814–821. 10.1038/nbt.2676 23975157 PMC3819121

[B33] Ley R. E. Peterson D. A. Gordon J. I. (2006). Ecological and evolutionary forces shaping microbial diversity in the human intestine. *Cell* 124 837–848. 10.1016/j.cell.2006.02.017 16497592

[B34] Lisko D. J. Johnston G. P. Johnston C. G. (2017). Effects of dietary yogurt on the healthy human gastrointestinal (GI) microbiome. *Microorganisms* 5:E6. 10.3390/microorganisms5010006 28212267 PMC5374383

[B35] Lozupone C. A. Hamady M. Kelley S. T. Knight R. (2007). Quantitative and qualitative beta diversity measures lead to different insights into factors that structure microbial communities. *Appl. Environ. Microbiol.* 73 1576–1585. 10.1128/aem.01996-06 17220268 PMC1828774

[B36] Lozupone C. A. Knight R. (2015). The UniFrac significance test is sensitive to tree topology. *BMC Bioinformatics* 16:211. 10.1186/s12859-015-0640-y 26150095 PMC4492014

[B37] Macfarlane S. Macfarlane G. T. (2003). Regulation of short-chain fatty acid production. *Proc. Nutr. Soc.* 62 67–72. 10.1079/pns2002207 12740060

[B38] Marco M. L. Heeney D. Binda S. Cifelli C. J. Cotter P. D. Foligné B. (2017). Health benefits of fermented foods: microbiota and beyond. *Curr. Opin. Biotechnol.* 44 94–102. 10.1016/j.copbio.2016.11.010 27998788

[B39] May L. A. (2011). *Saline-Saturated DMSO-EDTA as A Storage Medium for Microbial DNA Analysis from Coral Mucus Swab Samples.* Silver Spring: NOAA.

[B40] Penas-Steinhardt A. Barcos L. S. Belforte F. S. de Sereday M. Vilariño J. Gonzalez C. D. (2012). Functional characterization of TLR4 3725 G/C polymorphism and association with protection against overweight. *PLoS One* 7:e50992. 10.1371/journal.pone.0050992 23239997 PMC3519812

[B41] Petruzzelli M. Moschetta A. (2010). Intestinal ecology in the metabolic syndrome. *Cell Metab.* 11 345–346. 10.1016/j.cmet.2010.04.012 20444415

[B42] Rodríguez-Carrio J. López P. Sánchez B. González S. Gueimonde M. Margolles A. (2017). Intestinal dysbiosis is associated with altered short-chain fatty acids and serum-free fatty acids in systemic lupus erythematosus. *Front. Immunol.* 8:23. 10.3389/fimmu.2017.00023 28167944 PMC5253653

[B43] Roesch L. F. W. Casella G. Simell O. Krischer J. Wasserfall C. H. Schatz D. (2009). Influence of fecal sample storage on bacterial community diversity. *Open Microbiol. J.* 3 40–46. 10.2174/1874285800903010040 19440250 PMC2681173

[B44] Rognes T. Flouri T. Nichols B. Quince C. Mahé F. (2016). VSEARCH: a versatile open source tool for metagenomics. *PeerJ.* 4:e2584. 10.7717/peerj.2584 27781170 PMC5075697

[B45] Schmidt M. I. Duncan B. B. Vigo A. Pankow J. S. Couper D. Ballantyne C. M. (2006). Leptin and incident type 2 diabetes: risk or protection? *Diabetologia* 49 2086–2096. 10.1007/s00125-006-0351-z 16850292

[B46] Schnorr S. L. Candela M. Rampelli S. Centanni M. Consolandi C. Basaglia G. (2014). Gut microbiome of the Hadza hunter-gatherers. *Nat. Commun.* 5:3654. 10.1038/ncomms4654 24736369 PMC3996546

[B47] Schultze A. Akmatov M. K. Andrzejak M. Karras N. Kemmling Y. Maulhardt A. (2014). Comparison of stool collection on site versus at home in a population-based study. *Bundesgesundheitsblatt - Gesundheitsforschung - Gesundheitsschutz.* 57 1264–1269. 10.1007/s00103-014-2051-z 25293889 PMC4210724

[B48] Segata N. Izard J. Waldron L. Gevers D. Miropolsky L. Garrett W. S. (2011). Metagenomic biomarker discovery and explanation. *Genome Biol.* 12:R60. 10.1186/gb-2011-12-6-r60 21702898 PMC3218848

[B49] Sinha R. Chen J. Amir A. Vogtmann E. Shi J. Inman K. S. (2016). Collecting fecal samples for microbiome analyses in epidemiology studies. *Cancer Epidemiol. Biomark. Prev.* 25 407–416. 10.1158/1055-9965.EPI-15-0951 26604270 PMC4821594

[B50] Sommer F. Anderson J. M. Bharti R. Raes J. Rosenstiel P. (2017). The resilience of the intestinal microbiota influences health and disease. *Nat. Rev. Microbiol.* 15 630–638. 10.1038/nrmicro.2017.58 28626231

[B51] Surana N. K. Kasper D. L. (2017). Moving beyond microbiome-wide associations to causal microbe identification. *Nature* 552 244–247. 10.1038/nature25019 29211710 PMC5730484

[B52] Suzuki Y. Ikeda K. Sakuma K. Kawai S. Sawaki K. Asahara T. (2017). Association between yogurt consumption and intestinal microbiota in healthy young adults differs by host gender. *Front. Microbiol.* 8:847. 10.3389/fmicb.2017.00847 28553274 PMC5425481

[B53] Tercera encuesta nacional de factores de riesgo para enfermedades no transmisibles (2013). *Tercera Encuesta Nacional de Factores de Riesgo Para Enfermedades No Transmisibles*. Available at: http://www.msal.gob.ar/images/stories/bes/graficos/0000000544cnt-2015_09_04_encuesta_nacional_factores_riesgo.pdf.

[B54] The Human Microbiome Project Consortium (2012). Structure, function and diversity of the healthy human microbiome. *Nature* 486 207–214. 10.1038/nature11234 22699609 PMC3564958

[B55] Ussar S. Griffin N. W. Bezy O. Fujisaka S. Vienberg S. Softic S. (2015). Interactions between gut microbiota, host genetics and diet modulate the predisposition to obesity and metabolic syndrome. *Cell Metab.* 22 516–530. 10.1016/j.cmet.2015.07.007 26299453 PMC4570502

[B56] van den Elsen L. W. Poyntz H. C. Weyrich L. S. Young W. Forbes-Blom E. E. (2017). Embracing the gut microbiota: the new frontier for inflammatory and infectious diseases. *Clin. Transl. Immunol.* 6:e125. 10.1038/cti.2016.91 28197336 PMC5292562

[B57] Vogtmann E. Chen J. Amir A. Shi J. Abnet C. C. Nelson H. (2017). Comparison of collection methods for fecal samples in microbiome studies. *Am. J. Epidemiol.* 185 115–123. 10.1093/aje/kww177 27986704 PMC5253972

[B58] Voigt A. Y. Costea P. I. Kultima J. R. Li S. S. Zeller G. Sunagawa S. (2015). Temporal and technical variability of human gut metagenomes. *Genome Biol.* 16:73. 10.1186/s13059-015-0639-8 25888008 PMC4416267

[B59] Wesolowska-Andersen A. Bahl M. I. Carvalho V. Kristiansen K. Sicheritz-Pontén T. Gupta R. (2014). Choice of bacterial DNA extraction method from fecal material influences community structure as evaluated by metagenomic analysis. *Microbiome* 2:19. 10.1186/2049-2618-2-19 24949196 PMC4063427

